# Endoscopic surveillance of Lynch syndrome at a highly specialized center in Sweden: An observational study of interval colorectal cancer and individual risk factors

**DOI:** 10.3389/fonc.2023.1127707

**Published:** 2023-02-20

**Authors:** Nigin Jamizadeh, Sophie Walton Bernstedt, Adrianna Haxhijaj, Anna Andreasson, Jan Björk, Anna Forsberg, Ann-Sofie Backman

**Affiliations:** ^1^ Unit of Gastroenterology, Department of Medicine Huddinge, Karolinska Institutet, Stockholm, Sweden; ^2^ Division of Gastroenterology, Medical Unit Gastroenterology, Dermatovenereology and Rheumatology, Karolinska University Hospital, Stockholm, Sweden; ^3^ Division of Upper Gastrointestinal Diseases, Karolinska University Hospital, Stockholm, Sweden; ^4^ Hereditary Cancer Unit, Theme Cancer, Karolinska University Hospital, Stockholm, Sweden; ^5^ Division of Clinical Epidemiology, Department of Medicine Solna, Karolinska Institutet, Stockholm, Sweden; ^6^ Division of Gastroenterology, Department of Medicine, Ersta Hospital, Stockholm, Sweden

**Keywords:** Lynch syndrome (LS), colorectal cancer, interval cancer, surveillance, colonoscopy

## Abstract

**Introduction:**

Lynch syndrome (LS) is the most common hereditary cause of colorectal cancer (CRC). In order to detect CRCs amongst LS patients, regular colonoscopies are recommended. However, an international agreement on an optimal surveillance interval has not yet been reached. In addition, few studies have investigated factors that could potentially increase the CRC risk amongst LS patients.

**Aims:**

The primary aim was to describe the frequency of CRCs detected during endoscopic surveillance and to estimate the interval from a clean colonoscopy to CRC detection amongst LS patients. The secondary aim was to investigate individual risk factors, including sex, LS genotype, smoking, aspirin use and body mass index (BMI), on CRC risk amongst patients that develop CRC before and during surveillance.

**Material and methods:**

Clinical data and colonoscopy findings from 366 LS patients’ 1437 surveillance colonoscopies were collected from medical records and patient protocols. Logistic regression and Fisher’s exact test were used to investigate associations between individual risk factors and CRC development. Mann-Whitney U test was used to compare the distribution of TNM stages of CRC detected before surveillance and after index.

**Results:**

CRC was detected in 80 patients before surveillance and in 28 patients during surveillance (10 at index and 18 after index). During the surveillance programme, CRC was detected within 24 months in 65% of the patients, and after 24 months within 35% of the patients. CRC was more common amongst men, previous and current smokers, and the odds of developing CRC also increased with an increasing BMI. CRCs were more often detected amongst *MLH1* and *MSH2* carriers during surveillance, compared to the other genotypes.

**Conclusions:**

We found that 35% of the CRC cases detected during surveillance were found after 24 months. *MLH1* and *MSH2* carriers were at higher risk of developing CRC during surveillance. Additionally, men, current or previous smokers, and patients with a higher BMI were at higher risk of developing CRC. Currently, LS patients are recommended a “one-size-fits-all” surveillance program. The results support the development of a risk-score whereby individual risk factors should be taken into consideration when deciding on an optimal surveillance interval.

## 1 Introduction

Colorectal cancer (CRC) is the third most common cancer type diagnosed worldwide, after breast cancer and lung cancer. Causing approximately 1 million deaths annually, CRC is the second leading cause of cancer-related deaths in the world, following lung cancer (1). Genetic factors have a great impact on the risk of CRC development; up to 30% of CRCs have been estimated to have a familial component (2). Accounting for 3% of all CRCs, Lynch syndrome (LS) is the most common genetic cause of colorectal cancer. The risk of developing extracolonic cancers is higher amongst LS patients than non-LS patients (3). The most common extracolonic cancers amongst LS patients include endometrial cancer, gastric cancer, ovarian cancer, small bowel cancer, pancreas cancer, and cancer in the urothelial tract (4). LS is caused by germline mutations in the mismatch repair (MMR) genes *MLH1, MSH2, MSH6, PMS2*, and *EPCAM*, which are inherited in an autosomal dominant fashion (5).

Although it has been established that several behavioral and environmental factors are important risk modifiers for the development of CRC in the general population (6, 7), few studies have investigated the impact of lifestyle and individual factors on CRC risk amongst LS patients.

The association between smoking and CRC risk in LS is still somewhat uncertain. Pande et al. (8) conducted a retrospective cohort study of 752 LS patients and found no difference in CRC risk between ever- and never-smokers. When the ever-smokers were divided into current and previous smokers, an increased CRC risk was shown amongst current smokers, whereas previous smokers showed a decreased risk of CRC. Watson et al. (9) included smoking data from 360 LS patients in their retrospective study and found that tobacco users had a higher CRC risk than non-users. However, a limitation of their study was the lack of subcategories amongst tobacco users, as current smokers were not distinguished from previous smokers. In terms of BMI, several studies suggest that male sex combined with higher BMI leads to an increased CRC risk, whereas this association could not be found amongst females with higher BMI and LS (10–12).

Moreover, LS patients with different genotypes seem to have different risks of CRC development. For instance, some studies suggest that MLH1 carriers are at a higher risk of developing CRC than PMS2 carriers (13–15). Meanwhile, MLH1 and MSH2 carriers are estimated to have a similar risk for CRC, and MSH6 carriers present an intermediate risk in the genetic spectrum (16).

In order to detect CRCs amongst LS patients, regular colonoscopies are recommended. However, no international agreement on an optimal surveillance interval has yet been reached. Annual surveillance is recommended for MLH1 and MSH2 carriers in Australia (6) and every one to two years in the United States, starting from the age of 20–25 (5). Differences in surveillance intervals can also be observed in Europe, where the European Society of Gastrointestinal Endoscopy (ESGE) recommends biennial surveillance of asymptomatic LS patients (17). However, the recommended surveillance interval is every two to three years in Finland, every one to two years in the Netherlands, and annually in Germany (6). In Sweden, the recommended surveillance interval is every one to two years (18), and patients with LS in Stockholm undergo annual surveillance.

The life-saving effect of surveillance colonoscopies has been established by several studies. De Jong et al. (19) studied the surveillance interval program introduced in the Netherlands during the late 1980s. The mortality rate of CRC amongst LS patients was investigated before and after 1990, and the results showed a decrease in CRC mortality after 1990, supporting the efficiency of a one- to two-year surveillance program.

In a study published in 2019, Engel et al. (6) investigated the optimal surveillance interval amongst a total of 2747 MLH1, MSH2, and MSH6 carriers. Data were collected from 16,327 colonoscopies performed between 1984 and 2015. The study, which included LS patients from the Netherlands, Germany, and Finland, concluded that there was no significant difference in cumulative CRC incidence between the countries, although the recommended surveillance intervals varied from one to every three years.

Debate around the optimal interval for endoscopic surveillance of LS patients is ongoing: some studies suggest that shorter intervals are more beneficial (19), whilst others suggest no difference in CRC risk amongst LS patients in countries with different surveillance intervals (6). It is therefore of great importance to study the endoscopic surveillance intervals of LS patients further, whilst analyzing individual factors affecting their CRC incidence risk, to identify risk patients who may benefit from shorter surveillance intervals.

The primary aim of this study was to study the frequency of CRCs detected during endoscopic surveillance and to estimate the interval from a clean colonoscopy to CRC detection amongst LS patients. The secondary aim was to investigate individual risk factors, including sex, LS genotype, smoking, aspirin use and BMI, on CRC risk amongst patients who develop CRC before and during surveillance.

## 2 Materials and methods

### 2.1 Study design and subjects

A single-center, observational cohort study was conducted at Karolinska University Hospital in Stockholm, Sweden. The study considered LS patients with an MMR gene mutation who were followed at the Karolinska University Hospital from 1989 to April 2021. All MMR gene mutations were confirmed according to the InSight Variant Committee’s classification (20) or reported to be pathogenic by the hospital’s genetics department if the variant was unknown. Of 427 LS patients registered at the clinic, 366 were eligible for inclusion. Part of the cohort has previously been described (18).

After the MMR gene mutation had been confirmed, patients were recommended an index colonoscopy within 3 months. If the colonoscopy was “clean”, surveillance continued with a recommended interval of 1-2 years.

CRCs were classified as detected before surveillance if they had been detected before the MMR gene mutation was confirmed. A CRC detected at index or after index (where index is defined as the first colonoscopy after LS diagnosis is given, and after index is defined as the second colonoscopy after the MMR gene mutation is confirmed) was classified as detected during surveillance. Patients with multiple CRCs, detected both before and during surveillance, are also included in the “during surveillance” group below. Colonoscopies were counted and analyzed up until CRC detection. To study interval cancers only, the colonoscopies detecting CRC had to follow a “clean” examination within a reasonable timeframe. Accordingly, index CRCs were excluded from the TNM distribution and surveillance interval calculations. Underweight, normal weight, overweight and obese were defined as BMI (<18.5 kg/m^2^), (18.5–24.9 kg/m^2^), (18.5-<25.0 kg/m^2^) and (25.0-<30.0 kg/m^2^), respectively.

### 2.2 Data collection

Data were collected from medical records retrospectively and structured patient protocols prospectively during patient consultations. Questions about sex, age, height, weight, smoking habits, aspirin use and previous cancer(s) were answered in the protocol at the first gastroenterology outpatient visit after the LS diagnosis was ascertained. Data from the medical records included colonoscopy results from the patients’ surveillance colonoscopies performed from 1989 to 2021. Additional data regarding the date of LS and CRC diagnosis, cause of LS and CRC diagnosis, LS genotype, TNM stage and localization of CRC, and the surgical procedure performed were collected from the medical records from 1975 to 2021.

### 2.3 Statistical analysis

Descriptive statistics are presented as numbers (n) and percentages (%) for categorical variables and as mean values and standard deviations (SD) for continuous variables. Univariable and multivariable logistic regression was used to investigate the associations between individual factors, including sex, BMI, smoking, aspirin use, LS genotype, and CRC development. Univariable logistic regression was used to test differences in sex, BMI, age at LS and CRC diagnosis, and smoking status between patients with CRC detected before and during surveillance, Fisher’s exact test was used to test differences in LS genotypes between the two groups. The Mann–Whitney U test was used to compare the distribution of TNM stages of CRC detected before and during surveillance. Statistical significance was set at p ≤0.05. All statistical calculations were performed in SPSS package 28 (IBM® SPSS Statistics® version 28) made for macOS.

### 2.4 Ethical considerations

Data were obtained from medical records and structured protocols filled in by the patients at the hospital. All the procedures being performed were part of the routine clinical care. Ethical approval was granted by the Regional Ethics Review Board in Stockholm, Sweden, with approval number 2017/2013-31/2 and the Swedish Ethical Review Authority with approval number 2022-00119-0.

## 3 Results

Of a total number of 1887 colonoscopies, 76% were performed within 24 months. Of these, 1437 were surveillance colonoscopies, registered up until the latest CRC detection.

There were 366 patients in the cohort, of which 108 had at least one CRC diagnosis (referred to as “CRC cohort” below). Of these, 80 had their CRC detected before the MMR gene mutation had been confirmed. Twenty-eight patients had their CRC detected during surveillance, of which ten had a CRC detected at index, eighteen after index and four had a CRC detected both before and during surveillance.

The patient characteristics of the study population in total, for those who developed CRC, and for those who had not developed CRC are presented in **Table 1**. In the total cohort, the mean age for LS diagnosis was 42 years, and the most common LS genotype was MLH1 (45%), followed by MSH2 (28%). More than half the total cohort were never-smokers (57%).

**Table 1:** Patient characteristics in total and separated by CRC status.

Variable	Total cohort (n=366) n (%)	CRC cohort (n=108) n (%)	Non-CRC cohort (n=258) n (%)
Sex			
Men	169 (46)	59 (55)	110 (43)
Women	197 (54)	49 (45)	148 (57)
Deceased	15 (4)	8 (7)	7 (3)
Age at death (mean ± SD)	63.9 ± 15.2	67.8 ± 14.8	59.4 ± 15.5
Genotype			
* MLH1*	164 (45)	55 (51)	109 (42)
* MSH2*	103 (28)	33 (30)	70 (27)
* MSH6*	51 (14)	12 (11)	39 (15)
* PMS2*	38 (10)	6 (6)	32 (12)
* EPCAM*	6 (2)	1 (1)	5 (2)
Mixed genotype	4 (1)	1 (1)	3 (1)
Age at diagnosis (mean ± SD)	42.0 ± 15.4	49.2 ± 13.2	39.1 ± 15.2
Smoking status			
Current smoker	36 (10)	15 (14)	21 (8)
Previous smoker	104 (28)	42 (39)	62 (24)
Never-smoker	210 (57)	50 (46)	160 (62)
Missing data	16 (4)	1 (1)	15 (6)
Use of aspirin			
Current or previous	47 (13)	27 (25)	20 (8)
Never	295 (81)	77 (71)	218 (84)
Missing data	24 (7)	4 (4)	20 (8)
BMI (mean ± SD)	25.4 ± 4.6	26.3 ± 5.1	25.0 ± 4.4
Age at CRC diagnosis (mean ± SD)		45.5 ± 12.6	

Data are presented as numbers and percentages for nominal variables and as mean values and standard deviations for continuous variables. BMI, Body Mass Index; CRC, Colorectal Cancer; SD, Standard Deviation.

The logistic regression models investigating the association between potential risk factors and CRC development are presented in **Table 2**. In the univariable logistic regression, men had 62% higher odds of developing CRC than women, and patients with a PMS2 gene mutation had lower odds of developing CRC than MLH1 gene carriers (OR=0.37, 95% CI: 0.15–0.94). Smokers, both current and previous, had more than double the odds of patients who had never smoked. The odds of developing CRC also increased with an increasing BMI (OR=1.06, 95% CI: 1.01–1.12), whereas no significant difference could be found when comparing the BMI categories of underweight, overweight, and obese with normal BMI. Patients with current or previous aspirin use showed a significantly increased risk for CRC development than those who had never used it (OR=2.73, 95% CI: 1.51-4.94). The results from the multivariable logistic regression analysis demonstrate the same pattern, with the odds of developing CRC being significantly higher in men than women (OR=1.76, 95% CI: 1.08–2.87) as well as amongst current (OR=2.70, 95% CI: 1.21–6.03 and previous (OR=2.12, 95% CI: 1.26–3.59) smokers than never-smokers. Similarly, PMS2 carriers had lower odds of developing CRC than MLH1 carriers (OR=0.31, 95% CI: 0.12–0.81), and the results for BMI were identical in both logistic regression analyses. Additionally, patients with aspirin use had higher odds of CRC development than never-users.

**Table 2:** Risk factors for developing CRC.

Variable	Crude OR (95% CI)	*p*	Adjusted OR (95% CI)	*p*
Sex				
Men	1.62 (1.03-2.55)	0.04*	1.76 (1.08-2.87)	0.02*
Women	Ref	Ref	Ref	Ref
Genotype				
* MLH1*	Ref	Ref	Ref	Ref
* MSH2*	0.93 (0.55-1.58)	0.93	0.79 (0.45-1.38)	0.41
* MSH6*	0.61 (0.30-1.26)	0.18	0.51 (0.24-1.09)	0.08
* PMS2*	0.37 (0.15-0.94)	0.04*	0.31 (0.12-0.81)	0.02*
* EPCAM*	0.40 (0.05-3.48)	0.40	0.32 (0.03-3.19)	0.34
Mixed genotype	0.66 (0.07-6.50)	0.72	0.46 (0.05-4.64)	0.51
Smoking status				
Never-smoker	Ref	Ref	Ref	Ref
Previous smoker	2.17 (1.31-3.60)	<0.001*	2.12 (1.26-3.59)	0.05*
Current smoker	2.30 (1.10-4.77)	0.03*	2.70 (1.21-6.03)	0.02*
Use of aspirin				
Never	Ref	Ref	Ref	Ref
Current or previous	2.73 (1.51-4.94)	<0.001*	2.66 (1.41-5.01)	0.002*
BMI (linear)	1.06 (1.01-1.12)	0.01*	1.06 (1.01-1.12)	0.03*

BMI, Body Mass Index; CI, Confidence Interval; OR, Odds Ratio; Ref, Reference Variable. *Statistically significant values are marked with an asterisk. Univariable (crude OR) and multivariable (adjusted OR) logistic regression.

Patient characteristics of the CRC cohort in total and separated for cancer detection before surveillance (n=80) and during surveillance (n=28) are presented in **Table 3A**. Univariable logistic regression analysis was used to investigate the associations between individual factors and CRC detection during surveillance as an outcome. There was no statistically significant difference in sex distribution between the “before surveillance” group and the “during surveillance” group. Due to the low numbers in some of the genotypes, MLH1 and MSH2 were compared to the MSH6, PMS2, EPCAM, and mixed genotype between CRCs detected before and during surveillance. Only MLH1 and MSH2 carriers developed CRCs during surveillance, whereas MSH6, PMS2, EPCAM carriers and patients with a mixed genotype did not develop CRC during surveillance (p=0.01). The age at LS diagnosis was significantly lower amongst patients with CRC detection during surveillance than before surveillance (OR=0.95, 95% CI: 0.91–0.98), while no statistical difference was found in the age at CRC diagnosis between patients with a CRC detection before surveillance (44.9 ± 12.3) and during surveillance (47.5 ± 13.6).

**Table 3A:** Univariable logistic regression and Fisher’s exact test on patient characteristics of CRC cohort before and during surveillance.

Variable	Before surveillance (n=80) n (%)	During surveillance (n=28) n (%)	Crude OR (95% CI)	*p*
Sex				
Female	36 (73)	13 (27)	Ref	Ref
Male	44 (75)	15 (25)	1.1 (0.45-2.51)	0.90
Deceased	5 (63)	3 (37)		
Age at death (mean ± SD)	66.6 ± 17.0	69.7 ± 13.3		
Genotype**				
* MLH1*	32 (58)	23 (42)		0.01*
* MSH2*	28 (85)	5 (15)		0.01*
* MSH6*	12 (100)	0 (0)		Ref
* PMS2*	6 (100)	0 (0)		Ref
* EPCAM*	1 (100)	0 (0)		Ref
Mixed genotype	1 (100)	0 (0)		Ref
Age at diagnosis (mean ± SD)	51.4 ± 13.1	42.9 ± 11.5	0.95 (0.91–0.98)	0.05*
Smoking status				
Current smoker	9 (60)	6 (40)	1.90 (0.57–6.37)	0.30
Previous smoker	33 (79)	9 (21)	0.78 (0.29–2.05)	0.61
Never-smoker	37 (74)	13 (26)	Ref	Ref
Missing data	1 (100)	0 (0)		
BMI (mean ± SD)	26.1 ± 4.5	27.0 ± 6.7	1.03 (0.95-1.12)	0.42
Age at CRC diagnosis (mean ± SD)	44.9 ± 12.3	47.5 ± 13.6	1.02 (0.98-1.05)	0.35

Data are presented as numbers and percentages for nominal variables and as mean values and standard deviations for continuous variables. BMI, Body Mass Index; CI, Confidence Interval; OR, Odds Ratio; Ref, Reference Variable; SD, Standard Deviation. *Statistically significant values are marked with an asterisk. ** Genotypes in cursive and mixed genotype using Fisher’s exact test.

Of twenty-eight CRC cases detected during surveillance, ten CRC cases were detected at index and eighteen CRC cases were detected after index. The patient characteristics of the CRC cohort in total and separated for cancer detection before surveillance (n=80) and after index (n=18) are presented in **Table 3B**. There was a significant difference in LS genotype between the two groups, where only MLH1 and MSH2 carriers had CRC detection after index (p<0.001). The age at LS diagnosis was significantly lower amongst patients with CRC detection after index than before surveillance (OR=0.95, 95% CI:0.91–0.98).

**Table 3B:** Univariable logistic regression and Fisher’s exact test on patient characteristics of CRC cohort before surveillance and after index.

Variable	Before surveillance (n=80) n (%)	After index (n=18) n (%)	Crude OR (95% CI)	*p*
Sex				
Female	36 (84)	7 (16)	Ref	Ref
Male	44 (80)	11 (20)	0.92 (0.39-2.18)	0.85
Deceased	5 (63)	3 (37)		
Age at death (mean ± SD)	66.6 ± 17.0	69.7 ± 13.3		
Genotype**				
* MLH1*	32 (70)	14 (30)		<0.001*
* MSH2*	28 (88)	4 (12)		<0.001*
* MSH6*	12 (100)	0 (0)		Ref
* PMS2*	6 (100)	0 (0)		Ref
* EPCAM*	1 (100)	0 (0)		Ref
Mixed genotype	1 (100)	0 (0)		Ref
Age at LS diagnosis (mean ± SD)	51.4 ± 13.1	41.9 ± 12.1	0.95 (0.91–0.98)	0.05*
Smoking status				
Current smoker	9 (75)	3 (25)	1.90 (0.57-6.37)	0.30
Previous smoker	33 (87)	5 (13)	0.78 (0.29-2.05)	0.61
Never-smoker	37 (79)	10 (21)	Ref	Ref
Missing data	1 (100)	0 (0)		
BMI (mean ± SD)	26.1 ± 4.5	26.5 ± 5.7	1.03 (0.95-1.12)	0.45
Age at CRC diagnosis (mean ± SD)	44.9 ± 12.3	49.3 ± 15.1	1.02 (0.98-1.05)	0.35

Data are presented as numbers and percentages for nominal variables and as mean values and standard deviations for continuous variables. BMI, Body Mass Index; CI, Confidence Interval; OR, Odds Ratio; Ref, Reference Variable; SD, Standard Deviation. *Statistically significant values are marked with an asterisk. ** MLH1 and MSH2 compared to MSH6, PMS2, EPCAM and mixed genotype using Fisher’s exact test.

TNM stages of the CRCs detected before surveillance and during surveillance are presented in **Table 4**. Of 108 CRC cases, data on TNM classification were available for 86 CRCs. Of 18 interval CRCs, 17 had a TNM classification. Before surveillance, 46% of the CRCs were classified as stage I and II, and after index, 67% of the CRCs were classified as stage I and II. No significant difference was found between the TNM stage distribution before surveillance compared to after index (p=0.5).

**Table 4:** Distribution of TNM classification between CRC detected before and during surveillance.

	Before surveillance	During surveillance		
TNM stage	n (%)	At index n (%)	After index n (%)	Total, n (%)
Stage I	17 (21)	8 (80)	9 (50)	34 (31)
Stage II	20 (25)	2 (20)	3 (17)	25 (23)
Stage III	21 (26)	0 (0)	3 (17)	24 (22)
Stage IV	2 (3)	0 (0)	2 (11)	4 (4)
Missing data	20 (25)	0 (0)	1 (6)	21 (19)
Total	80 (100)	10 (100)	18 (100)	108 (100)

Data are presented as numbers (n) and percentages (%).

**Figure 1:**
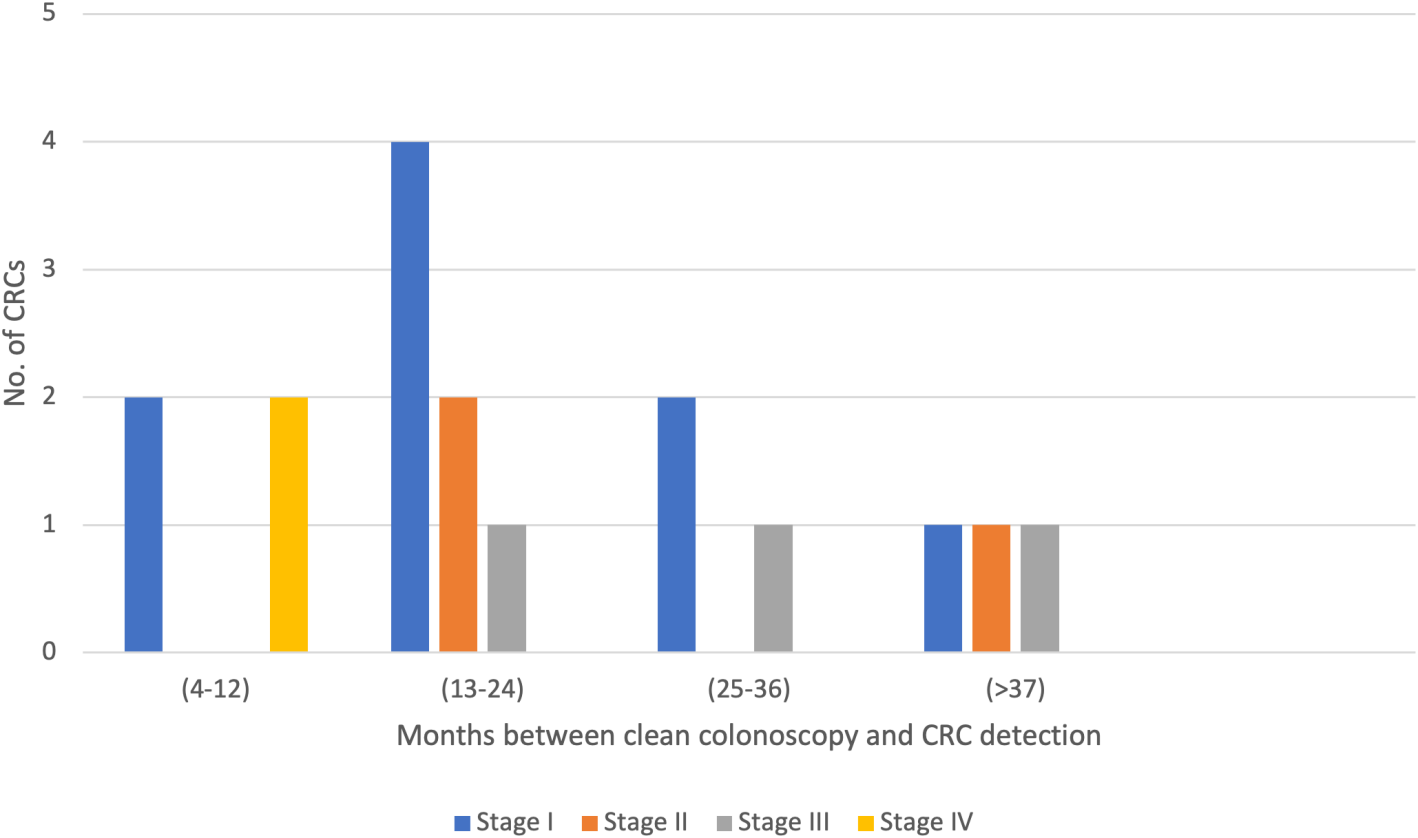
Colorectal cancer (CRC) detection after index. Intervals between clean colonoscopies and CRC detection of TNM-classified CRCs detected after index.

Surveillance intervals of the TNM-classified CRC cases found during surveillance are presented in **Figure 1**. Of the 17 CRC cases, 11 (65%) were detected within a 24-month interval. Two CRC cases were detected within 6 months, of which one had a shorter interval due to a suspect polyp with LGD, which turned out to be an adenocarcinoma. The second CRC case was detected due to post-surgical CRC symptoms, which turned out to be a relapse 6 months after the latest clean colonoscopy.

## 4 Discussion

In this study, we aimed to describe the frequency of CRCs detected during surveillance colonoscopies and estimate the interval from a clean colonoscopy to CRC detection amongst LS patients. We also aimed to evaluate individual risk factors, including sex, LS genotype, smoking, aspirin use and BMI, on CRC risk amongst patients who develop CRC before and during surveillance.

The results show that 80 of 108 CRC cases were detected before surveillance, and twenty-eight CRC cases were detected during surveillance. Of these, ten were index CRCs and eighteen were interval CRCs. Most of the CRC cases detected during surveillance were detected within a 24-month interval. We found that the risk of CRC detection during surveillance is significantly higher amongst MLH1 and MSH2 carriers than among MSH6, PMS2, and EPCAM carriers and patients with a mixed genotype. When studying individual risk factors, we found that current and previous smokers had almost twice as high odds of developing CRC as non-smokers, and patients who were male and had a higher BMI had higher odds of CRC development than women and patients with a lower BMI.

The ESGE’s surveillance guidelines for asymptomatic LS patients recommends a surveillance interval of 24 months (17). Our results show that 65% of the CRC cases detected after index were detected within this interval (**Figure 1**). Around 35% of the CRC cases were detected within 24–37 months or longer, which could be explained by poor patient compliance or the organization’s failure to adhere to hospital routines, leading to the postponement of colonoscopy appointments.

Contrary to both our initial expectations and a previous study by Engel et al. (21), we could not find a significant difference in TNM stage distribution between CRC cases detected before surveillance and after index. One possible explanation for this could be the small number of TNM-classified CRCs in the medical records of the LS patients. We did not detect a difference in TMN classification amongst CRCs detected within different colonoscopy intervals either. However, this result should be interpreted with caution as colonoscopy findings are subjective; therefore, a missed lesion at a “clean colonoscopy” could lead to a CRC with a higher TNM classification after a shorter interval.

In terms of risk factors, we found that men have a higher risk of developing CRC than women (**Table 2**). We also found that both previous and current smokers have higher odds of developing CRC than never-smokers, which is in line with the results Pande et al. presented in their study (9). In addition, our results show that an increase per BMI unit is associated with higher odds of CRC development, whereas no significant difference could be found within BMI categories of underweight, overweight, and obese when compared to normal BMI. This could partly be explained by the small sample size in the different BMI categories of the cohort. Previous studies have concluded that male sex combined with high BMI are risk factors for developing CRC, whereas this association could not be found amongst females with higher BMI and LS (11–13). We did not compare men and women when performing the logistic regression analysis on BMI. However, this could be of interest for future research.

Several randomized control trials have investigated the role of aspirin on adenoma recurrence, most of which have found a significant decrease in adenoma recurrence and CRC risk amongst aspirin-users (22–24). Interestingly, we found that patients with current or previous use of aspirin had higher odds of developing CRC than never-users. This could partly be explained by the time of data collection; the aspirin use could have been initiated as a secondary prevention after CRC diagnosis was given, leading to a larger proportion of aspirin-users in the CRC cohort. However, the indication for aspirin-use was not collected and is therefore not known.

Another important finding of this study is the difference between LS genotypes amongst patients with CRC detection before and during surveillance. The results show that MLH1 and MSH2 carriers have higher odds of CRC development during surveillance than MSH6, PMS2, and EPCAM carriers and patients with a mixed genotype. In accordance with these results, other studies have found that MLH1 and MSH2 carriers have a higher risk of developing CRC than MLH6 carriers (25), as well as PMS2 carriers (14–16), which could also be explained by the more rapid CRC development amongst MLH1 and MSH2 carriers (6). This finding could be used to bring forward a more individualized approach towards LS surveillance in which genotype is taken into consideration when an appropriate surveillance interval is recommended, which has previously been proposed by Goverde et al. (26).

This study is conducted on the outcome of colonoscopy surveillance amongst LS patients with CRC data, as well as structured data on individual factors, which are compared amongst patients with CRC detection before and during surveillance. Other studies, such as that conducted by Engel et al. (6), have investigated the optimal surveillance interval amongst LS patients but were unable to take individual risk factors into account.

A limitation of this study is the fact that it is a single-center study with results from one hospital only. Since genetic testing in Stockholm is only performed at the Karolinska University Hospital, however, most of the LS patients had their follow-up at this hospital as well. Another limitation of this study is the lack of quantification within the “smoking status” variable. It could be of benefit to collect data about smoking in pack-years to have clearer definitions within the categories of smokers. However, since our results show a significant increase of CRC development amongst current and previous smokers, this is an important finding to bring forward.

Another limitation of this study is that it revolves around quantitative data on colonoscopies, but does not take qualitative data into consideration. Lappalainen et al. suggested in their study that the quality of colonoscopy is usually not correlated with incident CRCs in LS (27). However, it could be of importance to take the quality of colonoscopies into account when investigating an optimal surveillance interval; the adenoma detection rate and cleanliness of bowel are important since incomplete colonoscopies might cause a delay in CRC detection. Therefore, the quality of the surveillance endoscopies performed in our cohort needs to be studied further.

In conclusion, we found that 35% of the CRC cases detected during surveillance were found after the ESGE’s recommended interval of 24 months (17). MLH1 and MSH2 carriers were at higher risk of developing CRC during surveillance. Additionally, men, current or previous smokers, and patients with a higher BMI were at higher risk of developing CRC. Currently, LS patients are recommended a “one-size-fits-all” colonoscopy surveillance program. The present results, however, support the development of a risk score in which individual risk factors, such as sex, genotype, smoking, and BMI, should be taken into consideration when identifying LS patients that may benefit from an annual surveillance program and individuals at low risk for whom frequency of surveillance may be reduced.

## Data availability statement

The data sets presented in this article are not readily available. The data set is still under construction for research purposes. Requests to access the data sets should be directed to ann-sofie.backman@ki.se.

## Author contributions

Study concept and design: A-SB. Acquisition of data: SB, NJ, JB, AH, and A-SB. Statistical analysis: NJ and AA. Drafting of the manuscript: NJ, AA, AF, and A-SB. All authors contributed to the article and approved the submitted version.
